# Role of HTLV-1 *orf*-*I* encoded proteins in viral transmission and persistence

**DOI:** 10.1186/s12977-019-0502-1

**Published:** 2019-12-18

**Authors:** Sarkis Sarkis, Veronica Galli, Ramona Moles, David Yurick, Georges Khoury, Damian F. J. Purcell, Genoveffa Franchini, Cynthia A. Pise-Masison

**Affiliations:** 1 Animal Models and Retroviral Vaccines Section, Vaccine Branch, National Cancer Institute, National Institutes of Health, Bethesda, MD USA; 2 Department of Microbiology and Immunology, The Peter Doherty Institute for Infection and Immunity, The University of Melbourne, Parkville, VIC Australia

**Keywords:** HTLV-1, STLV-1, *orf*-*I*, p12/p8, *rex*-*orf*-*I*, Adult T-cell leukemia/lymphoma, ATLL, HTLV-1 associated myelopathy/tropical spastic paraparesis, HAM/TSP, Immune evasion

## Abstract

The human T cell leukemia virus type 1 (HTVL-1), first reported in 1980 by Robert Gallo’s group, is the etiologic agent of both cancer and inflammatory diseases. Despite approximately 40 years of investigation, the prognosis for afflicted patients remains poor with no effective treatments. The virus persists in the infected host by evading the host immune response and inducing proliferation of infected CD4^+^ T-cells. Here, we will review the role that viral *orf*-*I* protein products play in altering intracellular signaling, protein expression and cell–cell communication in order to escape immune recognition and promote T-cell proliferation. We will also review studies of *orf*-*I* mutations found in infected patients and their potential impact on viral load, transmission and persistence. Finally, we will compare the *orf*-*I* gene in HTLV-1 subtypes as well as related STLV-1.

## Background

Human T-cell leukemia virus type-1 (HTLV-1) was discovered in 1980 in T-cells in a patient with cutaneous T-cell lymphoma [1, 2]. It is a member of the *Delta retrovirus* genus, alongside the closely related HTLV-2, -3, and -4 viruses, simian T-cell leukemia viruses (STLV) 1–4, and bovine leukemia virus (BLV) [3–5]. HTLV-1 infects approximately 5 to 10 million individuals worldwide with the highest endemic rates of infection in southern Japan, the Caribbean, Central and South America, Africa, Northeast Iran, Romania, Australia, and Melanesia [6]. HTLV-1 has seven reported subtypes (subtypes A to G), which are primarily contained to their respective geographic regions [6–14].

While the majority of infected individuals remain asymptomatic, a low percentage (2–5%) develop one of two major diseases after a long period of clinical latency: Adult T-cell leukemia/lymphoma (ATLL), a disease characterized by malignant proliferation of CD4^+^ T-lymphocytes, or HTLV-1-associated myelopathy/tropical spastic paraparesis (HAM/TSP), a neurodegenerative condition [15–18]. Additionally, HTLV-1 is associated with other clinical disorders including HTLV-1-associated arthropathy, HTLV-1-associated uveitis, infective dermatitis, polymyositis, and chronic pulmonary disorders [18–26].

The manner in which HTLV-1 maintains persistent infection is likely associated with its ability to evade the host immune response. Immune evasion may also be associated with the proliferation of infected cells, leading to high proviral loads that correlate with disease progression. A high viral DNA burden in peripheral blood mononuclear cells has been associated with ATLL development [27, 28] and is considered a risk factor for HAM/TSP development [28, 29], particularly when there is a higher virus level in the cerebrospinal fluid than in peripheral blood [30]. In addition, HTLV-1-infected individuals have been shown to have diverse immunological alterations, such as high levels of inflammatory cytokines, spontaneous T-cell proliferation, and cellular maturation [31–36].

Several lines of evidence indicate that the HTLV-1 *open reading frame*-*I* (*orf*-*I*) is linked to immune evasion and viral replication and persistence. Unlike Tax and Rex, the HTLV-1 regulatory *orf*-*I* gene products are not required for virus replication and for the immortalization of human primary T-cells in vitro [37–39]. It has been shown, however, that human T-cell lines immortalized with HTLV-1 molecular clones lacking *orf*-*I* grow less efficiently than their wild-type counterpart clones and are more dependent upon the concentration of interleukin-2 (IL-2) in the media [40–42]. In addition, *orf*-*I* was found to be essential for HTLV-1 infection and replication in non-human primates, though not in rabbits [43]. In this review, we discuss the role of *orf*-*I* in immune regulation and in the context of the various HTLV subtypes.

## HTLV-1A *orf*-*I*

### Protein structure

**Fig. 1:**
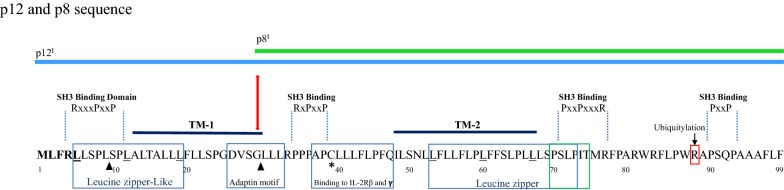
Structure of orf-I proteins p12 and p8. Amino acid sequence and putative functional domains of full length orf-I protein. The p12 protein is highly hydrophobic and contains an amino terminus noncanonical ER retention/retrieval motif (in bold), four putative proline-rich (PxxP) Src homology 3 (SH3)-binding domains, two putative leucine zipper (LZ) motifs, and an IL-2R β and γ chain binding motif (in blue boxes). The calcineurin-binding motif [^70^PSLP(I/L)T^75^] is indicated by a green box, and two transmembrane helices TM-1 and TM-2 domains are designated by black bars above the sequence. The black triangles indicate the two cleavage sites between amino acid positions 9 and 10, and 29 and 30, respectively. The asterisk denotes the position of cysteine 39. The proteolytic cleavage site G29/L30 leading to the production of p8 is indicated with a red arrow. The lysine-to-arginine variant is highlighted at position 88 by a red box. Arginine at this position increases the stability of the protein

The most studied *orf*-*I* gene is that of HTLV-1A, located at the 3′ end of the viral genome. It encodes the 99 amino acid p12 protein that can be proteolytically cleaved at the amino terminus to give rise to the p8 protein (Fig. 1) [44]. Amino acid sequence analysis of p12 predicts a noncanonical endoplasmic reticulum (ER) retention/retrieval signal between amino acids 1–5, two putative leucine zipper (LZ) motifs, two putative transmembrane domains between amino acids 12–30 and 48–67, a calcineurin-binding motif between amino acids 70–86, four putative proline-rich (PxxP) Src homology 3 (SH3)-binding domains, and a putative adaptin motif [45–47]. These structural features are thought to contribute to protein localization, dimerization, and protein–protein interactions. The naturally occurring p12 variant K88 is commonly found in HTLV-1 strains from HAM/TSP patients, while a second variant, R88, is found in virus strains from ATLL patients and healthy carriers [48]. R88 has much greater stability compared to K88, which is ubiquitinated and rapidly degraded by the proteasome [48]. Studies have found that p12 dimerization occurs through a disulfide bond at the conserved cysteine 39 residue of p12 and, when C39 is palmitoylated, the protein remains monomeric [49]. HTLV-1 strains containing either a serine (S39) or an arginine (R39) residue at this location have also been identified [50]. The actual importance of this cysteine residue to p12 function and regulation remains undetermined.

Computer program analysis of the p12 protein sequence predicted two cleavage sites which were verified by mutagenesis studies showing that p12 undergoes a stepwise posttranslational proteolytic cleavage [46]. The first cleavage occurs between amino acids 9 and 10 and is followed by a second cleavage between amino acids 29 and 30 [46]. While the first cleavage between amino acids 9 and 10 removes the endoplasmic reticulum (ER) retention/retrieval signal of p12, cleavage between amino acids 29 and 30 generates the p8 protein (Fig. 1) [46]. Interestingly, while mutation analysis shows that cleavage first occurs at L9/10S, cleavage at the second site (G29/30L) follows so quickly afterward that detection of the first cleavage product is not seen or rarely seen in expression systems. In addition, later studies show that variation in the amino acid sequence influences protein cleavage and the abundance of p12 compared to p8. The p12 protein localizes to cellular endomembranes, particularly within the ER and Golgi apparatus, while p8 traffics to lipid rafts at the cell surface and is recruited to the immunological synapse upon T-cell receptor (TCR) ligation [46, 51–53]. The only protein so far identified to have amino acid similarity to p12 is the bovine papillomavirus (BPV)-transforming E5 protein, but E5 does not carry putative SH3 binding motifs [54, 55].

Indirect evidence suggests that infected individuals express *orf*-*I*-encoded proteins. The singly spliced mRNA encoding p12/p8 has been detected in ex vivo HTLV-1-infected T-cells and macrophages [44]. Moreover, sera from humans and rabbits infected with HTLV-1 contain antibodies capable of detecting overexpressed or recombinant p12 [56]. Cytotoxic T-lymphocyte (CTL) responses to *orf*-*I* products have also been detected in HTLV-1-infected individuals [57]. While the p12/p8 proteins are highly conserved, several variants have been identified [58]. Of these, G29S, P34L, S63P, R88K and S91P were the most frequent non-synonymous mutations observed. When present, G29S, P34L, and S63P were found to express a non-cleavable p12, whereas the rare D26N and D26E mutations predominantly expressed p8 [58]. Interestingly, the authors found that the pattern of p12 and p8 expression correlated with proviral load [58]. In a second study using a computational approach to examine p12/p8 sequence variants (D26N, G29S, P34L, L40F, P45L, S63P, L66P, S69G, R83C) in healthy carriers of HTLV-1 and HAM/TSP patients, P45L, S69G, and R88K were found more frequently in patients positive for HAM/TSP, and D26N, P34L, C39R, F61L, and R83C were found to be associated with low proviral load [59].

### p12/p8 in T-cell proliferation

#### IL-2 receptor activation and STAT5 signaling

**Fig. 2:**
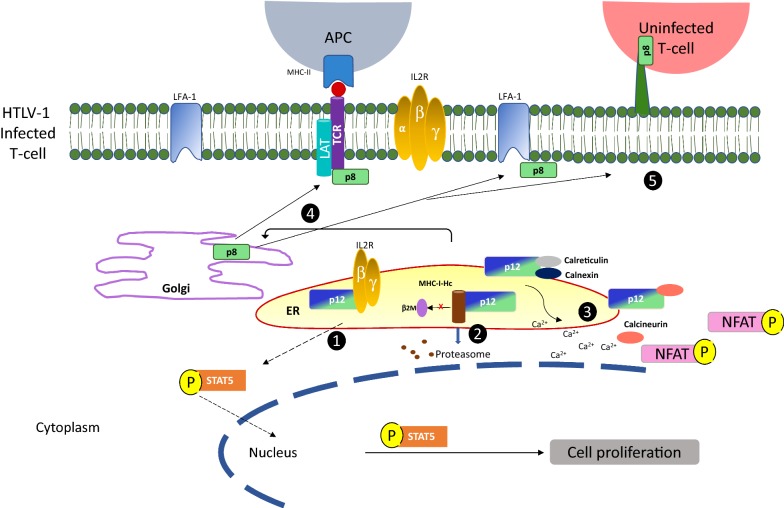
p12/p8 protein trafficking and function. (1) In the endoplasmic reticulum (ER), p12 binds to and retains the immature forms of interleukin-2 receptor (IL-2R) β and γ subunits, decreasing their trafficking to the plasma membrane. However, accumulation of the p12/IL-2R γ and β chains in the ER results in signal transducer and activator of transcription 5 (STAT5) phosphorylation in the absence of IL-2, allowing STAT5 translocation to the nucleus to promote cellular proliferation. (2) In the ER, p12 interacts with the immature heavy chains of MHC-I (MHC-I-Hc), binding to its α chain and preventing their interaction with β2 microglobulin (β2m). This leads to its degradation by the proteasome and decreased MHC-I expression at the cell surface. (3) HTLV-1A p12 also mediates the release of calcium ions (Ca^2+^) from the ER by binding calreticulin and calnexin. The release of Ca^2+^ inhibits the binding of calcineurin to the nuclear factor of T-cells (NFAT), preventing its dephosphorylation, nuclear translocation, induction of IL-2 expression and T-cell activation. In addition, p12 can inhibit the NFAT pathway by binding to calcineurin. (4) The p12 protein is proteolytically cleaved in the ER, leading to the formation of p8 that traffics to the cell surface. There, p8 increases T-cell adhesion through lymphocyte function-associated antigen-1 (LFA-1) clustering and promotes the formation of cell-to-cell contacts. (5) Further, p8 enhances the number and length of cellular conduits between T-cells, thereby enhancing signal transduction and HTLV-1 transmission

HTLV-1 persists primarily through expansion of infected cells, and while IL-2 promotes T-cell proliferation and controls immune responses [60], T-cells infected with HTLV-1 proliferate in the absence of IL-2. This IL-2 independence correlates with constitutive activation of the Janus-associated kinase and signal transducer and activator of transcription (JAK-STAT) pathway, a transcription factor cascade that affects cell proliferation, differentiation, and apoptosis [61]. Initially, p12 and p8 were not thought to have a role in IL-2 independence since they did not affect the expression of the interleukin-2 receptor (IL-2R) or the phosphorylation of JAK-STAT proteins [62]. However, later studies demonstrated that these proteins bind the β and γ_c_ chains of the immature IL-2R, stabilizing them in a pre-Golgi compartment and preventing their trafficking to the plasma membrane, leading to decreased IL-2R on the cell surface [63]. Co-immunoprecipitation experiments demonstrated that p12/p8 bind to a 20 amino acid region proximal to amino acid 350 of the IL-2R β chain that is critical for JAK1 and JAK3 recruitment [41]. The interaction of p12/p8 with the immature IL-2R leads to an increase in signal transducer and activator of transcription 5 (STAT5) phosphorylation and DNA binding activity in the absence of IL-2 [41]. Thus, the binding of p12/p8 to IL-2R allows T-cells to reproduce in the absence of IL-2 and with suboptimal antigen stimulation, providing HTLV-1-infected cells with a noteworthy proliferative advantage (Fig. 2) [41].

#### Calcium signaling and NFAT activation

The p12 protein localizes to the endoplasmic reticulum [53], where it is able to mediate an increase in cytosolic calcium (Ca^2+^) through its interaction with the calcium-binding proteins calreticulin and calnexin [51]. The presence of p12 in T-cells increases calcium release from the ER through inositol trisphosphate receptors and the facilitation of capacitive calcium entry through Ca^2+^ channels at the plasma membrane in response to the lowered ER calcium content (Fig. 2) [64, 65]. By depleting ER calcium stores and increasing cytosolic calcium, p12 is able to modulate a range of processes including T-cell proliferation, viral replication, and viral spread.

Early studies demonstrated that p12 can increase T-cell proliferation by activating the nuclear factor of activated T-cells (NFAT), which depends on calcium-binding proteins for dephosphorylation and nuclear import (Fig. 2) [64–66]. NFAT proteins play several important roles in regulating T-cell activity and are involved in their regulation, differentiation, self-tolerance, and in controlling thymocyte development (reviewed in [67]). NFAT can be activated through a complex TCR signaling cascade: following TCR engagement at the cell surface, the Lck and Fyn protein tyrosine kinases phosphorylate TCRζ and the CD3 subunits, allowing ZAP70 to dock to these phosphorylated domains. Activated ZAP70 phosphorylates the linker for activation of T-cells (LAT), which in turn binds and activates phospholipase C-γ-1 (PLCγ1) and leads to the production of inositol-1,4,5-trisphosphate and the release of Ca^2+^ from ER calcium stores. With the increase in cytosolic calcium, calmodulin and calcineurin are activated to dephosphorylate NFAT and permit its nuclear import. By modulating the regulation of cytosolic calcium levels, p12 mediates NFAT activation independent of the proximal TCR signaling molecules LAT and PLCγ1 [64]. Since NFAT binds the IL-2 promoter to increase transcription, the expression of p12 in T-cells thus supports IL-2 production in a calcium dependent manner [65].

However, p12 has also been shown to bind calcineurin, and its calcineurin-binding motif [^70^PSLP(I/L)T^75^] is homologous to that of NFAT [PXIXIT] [66]. The p12 protein might, therefore, act as a negative regulator of NFAT activation by competing with NFAT for calcineurin binding. The calcineurin binding motif is present in both p12 and p8, but it is currently not known whether p12/p8 homodimers or p12/p8 heterodimers bind calcineurin. Additional studies have revealed that p8, which localizes at the cell surface, is also capable of downregulating NFAT activity, though in a LAT-dependent manner [68]. Besides NFAT, p12 expression influences other calcium-regulated proteins, like the transcriptional coactivator p300 [69], which can in turn modulate the transcription of viral genes from the HTLV-1 LTR [70]. Moreover, p12 can promote intercellular viral spread by inducing lymphocyte function-associated antigen 1 (LFA-1) clustering on T-cells through a calcium-dependent mechanism (Fig. 2) [71].

#### p12/p8 and vacuolar ATPase

As suggested by their interaction with the H^+^ vacuolar ATPase, p12/p8 may affect signaling. The amino acids of HTLV-1 p12/p8 were found to be similar to those of the Bovine Papilloma Virus (BPV) E5 proteins. Like the BPV E5 oncoprotein [72, 73], p12/p8 interact with the 16 kDa subunit of the V-ATPase [54, 55]. While the transmembrane domains of p12/p8 appear to be unnecessary in V-ATPase binding, the proline-rich domain between amino acids 36 and 48 strengthens the bond [54, 55]. V-ATPase is found in clathrin-coated vesicles, lysosomes, endosomes, Golgi vesicles, endoplasmic reticulum, and synaptic vesicles, where it regulates the acidification of these intracellular vesicles [74]. By binding with viral proteins such as HTLV-1 p12/p8 and BPV E5, the proton pump may potentially interfere with functions such as receptor-ligand dissociation and protein trafficking within the endosomal/lysosomal compartment, but acidification remains essential for the formation of endosome carrier vesicles, which are intermediates between early and late endosomes [75, 76]. HTLV-1 is known to infect dendritic cells, and the acidification of lysosomes could play an important role in regulating virus entry or egress [77–79]. Indeed, the ablation of *orf*-*I* expression impairs HTLV-1 replication in dendritic cells [43].

### p12/p8 in host immunity

#### orf-I and MHC class I degradation

The major histocompatibility complex class I (MHC-I) antigen presentation pathway plays a central role in developing host immunity against pathogens. MHC-I molecules are expressed on the surface of all nucleated cells and present peptides to the TCRs of cytotoxic T lymphocytes. Effector CD8^+^ T-cells specifically recognize viral peptides via the TCR to destroy infected cells. Consequently, many viruses have evolved proteins whose main function is to interfere with this pathway [80]. In MHC-I molecules, the heavy chain (Hc) is non-covalently bound to a non-glycosylated β_2_ microglobulin (β2M) protein, with MHC-I-Hc binding affinity to β2M enhanced when in the presence of peptide. In this case, MHC-I-Hc folds and assembles into the peptide-MHC-I-Hc-β2M complex in the lumen of the ER [81]. The p12 proteins bind to newly synthesized MHC-I-Hc prior to their association with the β_2_-microglobulin necessary to form a mature MHC-I complex (Fig. 2) [52]. Improperly assembled proteins are removed from the ER for degradation [82], and p12/MHC-I-Hc complexes are thus ubiquitinated and retrotranslocated to the cytosol for degradation by the proteasome, resulting in decreased MHC-I cell surface expression. Notably, p8 also co-precipitates with MHC-I, but the biological significance of this interaction is unclear.

**Fig. 3:**
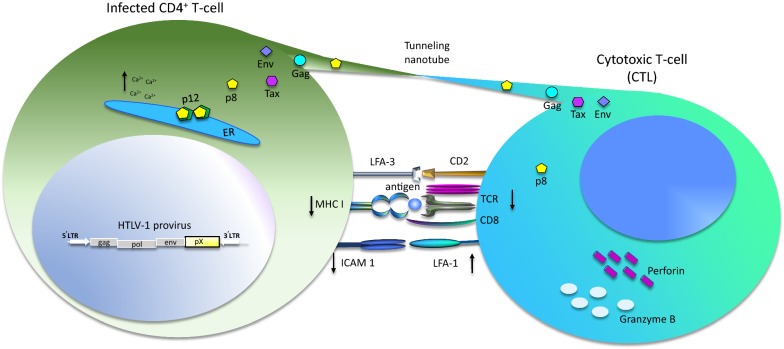
Effect of p12/p8 on cytotoxic T-cells (CTLs). Cytotoxic CD8 T-cells (CTL) recognize target cells bearing an appropriate antigen-MHC I complex via the T-cell receptor (TCR). CTLs carry out target cell killing by releasing the cytotoxic proteins, granzyme B and perforin. Both p12 and p8 expression are important for HTLV-1 inhibition of CTL killing. By inducing the proteasome degradation of immature MHC I, p12 decreases MHC I surface expression, reducing antigen presentation to CTLs. In addition, the reduction of ICAM-1 expression in infected cells further reduces cell adhesion. The p8 protein enhances the number and length of cellular conduits between T-cells, allowing for the transfer of target cell proteins to other cells, including p8 itself. Transferred p8 could alter intracellular signaling and dampen TCR signaling to inhibit CTL killing. The p8 protein also promotes T-cell adhesion through lymphocyte function-associated antigen-1 (LFA-1) clustering and by enhancing the formation of cell-to-cell contacts promoting viral transmission

*Orf*-*I* mRNA is expressed early after virus entry and is critical for establishing and maintaining viral infection in vivo [40, 43, 83, 84]. In a recent report comparing the expression of MHC-I on primary CD4^+^ T-cells infected with HTLV-1 molecular clones expressing neither p12 or p8 (p12KO), both p12 and p8 (WT), predominantly p8 (N26), or predominantly p12 (G29S), a decrease in surface MHC-I was only observed in the CD4^+^ T-cells infected with the G29S virus [58]. However, it must be noted that expression of both p8 and p12 was necessary to fully protect the infected cells from CTL killing (Fig. 3) [58]. Thus, the suppression of MHC-I antigen presentation by p12/p8 may allow HTLV-1 to evade adaptive immune surveillance in vivo and contribute to the expansion and accumulation of infected CD4^+^ T-cell clones over time.

#### orf-I and NK cell recognition

**Fig. 4:**
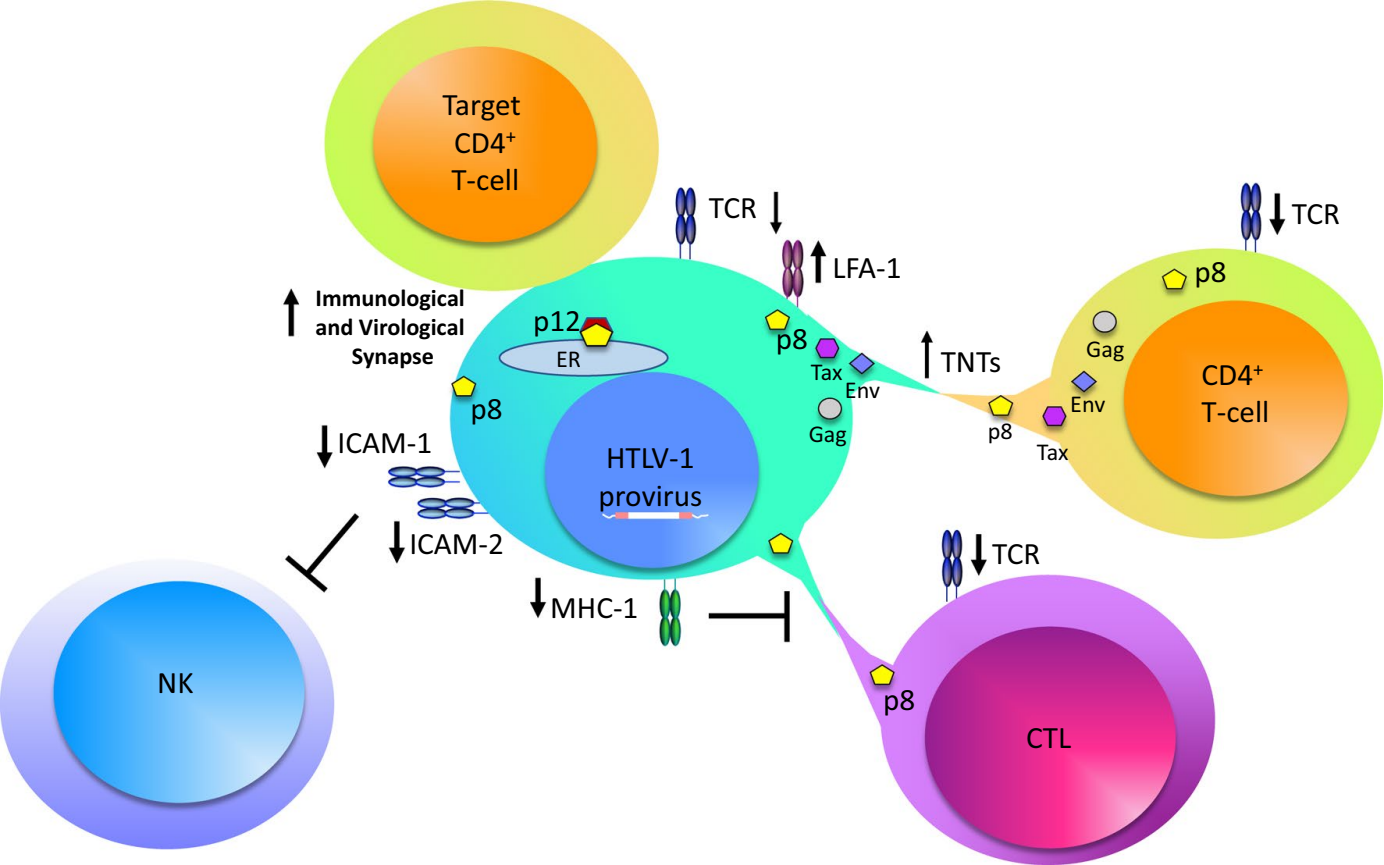
Impact of p12/p8 proteins on the host immune response. Summary of p12 and p8 influence on the host immune response to HTLV-1-infected cells. Expression of HTLV-1 p12/p8 results in decreased intercellular adhesion molecules (ICAM) and MHC-I expression, resulting in the inhibition of natural killer (NK) cell recognition and cytotoxic T-cell (CTL) killing. The p8 protein induces increased cell adhesion through increased lymphocyte function-associated antigen-1 (LFA-1) expression, increased virus transmission and cell signaling through tunneling nanotubes (TNTs) and virological synapse formation, and dampens T-cell receptor (TCR) signaling

While down modulation of MHC-I surface expression may allow infected cells to evade CTL recognition, it makes them susceptible to natural killer (NK) cell lysis. NK cells recognize and destroy cells that express low levels of MHC-I at their surface. Like CTLs, NK cells can kill infected cells directly by mediating cytolysis through perforin and granzyme production (reviewed in [85]). NK-target cell immune synapse is mediated by integrins such as leukocyte function antigen 1 (LFA-1) on the NK cell, and its ligand intercellular adhesion molecule 1 (ICAM-1) on the target cell. As shown in early studies, overexpression of the Tax protein increases the presence of adhesion molecules like LFA-3 and ICAM-1 [86, 87]. While high levels of ICAM-1 were found on transformed HTLV-1 cell lines that expressed Tax, the ligand was instead downregulated in several ATLL cell lines [87]. In more recent studies, a significant decrease in surface MHC-I and ICAM-1 and ICAM-2 (but not ICAM-3) was observed in primary CD4^+^ T-cells infected with HTLV-1 [88]. Furthermore, the infected cells in this study were shown to resist NK cell killing, which was moderately ameliorated by pretreatment of NK cells with IL-2 [88]. The majority of infected primary CD4^+^ T-cells did not express ligands for the NK cell activating receptors, natural cytotoxicity receptors, or NKG2D [88]. The authors did go on to show that *orf*-*I* expression was sufficient to decrease ICAM-1 and ICAM-2 expression in primary CD4^+^ T-cells. Treatment of Tax-expressing HTLV-1 transformed cells with pomalidomide (POM), an immunomodulatory drug used in the treatment of multiple myeloma [89], led to an increase in both surface MHC-I and ICAM-1. The effect of pomalidomide was shown to be *orf*-*I* dependent: MHC-I and ICAM-I expression increased in wild type (WT) HTLV-1 immortalized CD4^+^ T-cells after POM treatment, but their levels did not rise in HTLV-1 *orf*-*I* knockout immortalized CD4^+^ T-cells [90]. Thus, p12/p8 inhibit NK cell adhesion to T-cells and protect virus-infected cells from recognition in the presence of low levels of MHC-I (Fig. 4).

Interestingly, additional proteins with functions similar to that of p12 have been identified in HTLV-2 and STLV-3. For instance, the corresponding p10 protein is encoded by the *orf*-*I* region of HTLV-2, and an additional protein, p11, is encoded by the *orf*-*V* region, which overlaps with *orf*-*I*. Both proteins were shown to bind the MHC heavy chain, but do not bind the IL2Rβ chain or the 16-kDa subunit of the vacuolar H^+^ ATPase [91]. Moreover, STLV-3 p9 shares some structural and functional features with HTLV-1 p12. Despite a very low percent sequence similarity between the two proteins, STLV-3 p9 and HTLV-1 p12 showed identical localization patterns in transfected cells, and p9 expression promoted the downregulation of calreticulin signaling [92].

#### orf-I in virus transmission

Surface adhesion molecules like LFA and ICAM are also important in viral transmission. Cell-free virions are not easily detected in the blood plasma of infected individuals and cell-free virus is poorly infectious for most cell types except dendritic cells [77, 93–95]. HTLV-1 is transmitted through cell-to-cell contact via the virological synapse, biofilm-like extracellular viral assemblies, and cellular conduits [96–99]. The transfer of a virus through the virological synapse requires polarization of adhesion and cytoskeletal proteins to the point of cellular contact [96]. Recent evidence suggests that p8 enhances polysynapse formation and modulates LFA-1 clustering to increase the formation of cell to cell contact and facilitate virus transfer [71, 98]. In addition, p8 promotes the formation of thin membranous cellular conduits that allow intracellular communication between several different cell types (Figs. 3 and 4) [98, 100, 101]. Among these, p8 induces tunneling nanotubes (TNTs), thin structures containing F-actin and lacking tubulin that allow for communication between cells at a distance. Immune cells such as NK cells, macrophages, T-cells, and B-cells are known to interact through TNTs [102, 103], and the induction of TNTs by other viruses has been reported [104–108]. Through these structures, the HTLV-1 proteins Tax, Gag, and Env are transferred to target T-cells [98]. When HTLV-1-infected T-cells are treated with Cytarabine, a molecule shown to reduce TNT formation [109], virus production and transmission are shown to decrease [110].

Furthermore, the p8 protein was also shown to be transferred to target cells [98, 110]. Using a quantitative flow cytometry method, p8 was transferred to approximately 5% of recipient T-cells after 5 min of co-culture, in a process dependent on actin polymerization [111]. The presence of p8 was also shown to decrease T-cell activation by inhibiting proximal TCR signaling [68]. Upon ligation of the TCR to the major histocompatibility complex class II (MHC-II) of an antigen presenting cell, p8 localizes to the immunological synapse, where it decreases phosphorylation of LAT, PLCγ1, and Vav by a LAT-dependent mechanism [46, 68]. By dampening TCR signaling, p8 downregulates NFAT activation, which is a crucial pathway in T-cell activation [64, 68]. Induction of T-cell anergy, a state in which T-cells become unresponsive to TCR stimulation, results in decreased Tax activity and HTLV-1 replication [68]. Since it has recently been shown that p8 transfers to target cells, it is possible that p8 induces T-cell anergy in cells that neighbor HTLV-1-infected cells to expand opportunities to safely transfer the virus to target cells [98].

#### Role of p12/p8 in HTLV-1 infectivity in vivo

To more closely examine the role of p12 in the initial stages of infection, investigators used animal models to study HTLV-1 molecular clones [43, 83]. Before identification of HBZ [112, 113], it was reported that *orf*-*I* expression was necessary for HTLV-1 infection in the rabbit model [83]. In addition to the deletion of *orf*-*I* in those studies, however, the molecular clone used had a frameshift affecting the gene encoding HBZ (Additional file 1: Figure S1). Therefore, it is unclear whether the results were due to the deletion of *hbz*, *orf*-*I*, or both. A more recent study using an HTLV-1 molecular clone which selectively disrupted *orf*-*I* expression revealed that *orf*-*I* is essential for infectivity in the macaque model, but not in the rabbit model [43]. Moreover, the finding that p12 is required for viral infectivity in macaques was found to be related to its role in supporting HTLV-1 infectivity of dendritic cells in vitro [43]. Additional i*n vivo* studies in macaques have provided further support for the notion that p8 and p12 are important for viral persistence and spread [58]. When these molecular clones were used in a humanized mouse model, wild type HTLV-1 virus caused a polyclonal expansion of infected CD4^+^CD25^+^ T-cells. Notably, when the p12 knockout virus was used instead, infection only occurred after the virus reverted to wild type [84]. These studies suggest that maintaining p12/p8 expression is important for enabling viral infection and persistence. This is in line with results on HTLV-2 in the rabbit model. The authors showed that the sequences at the 3′ end of the proximal portion of HTLV-2, corresponding to the p12 region in HTLV-1, are not necessary for infection, but confer increased replicative capacity in vivo [114].

### *orf*-*I* genetic variation of HTLV-1 subtypes

#### Genetic variation in HTLV-1A and HTLV-1C orf-I

**Fig. 5:**
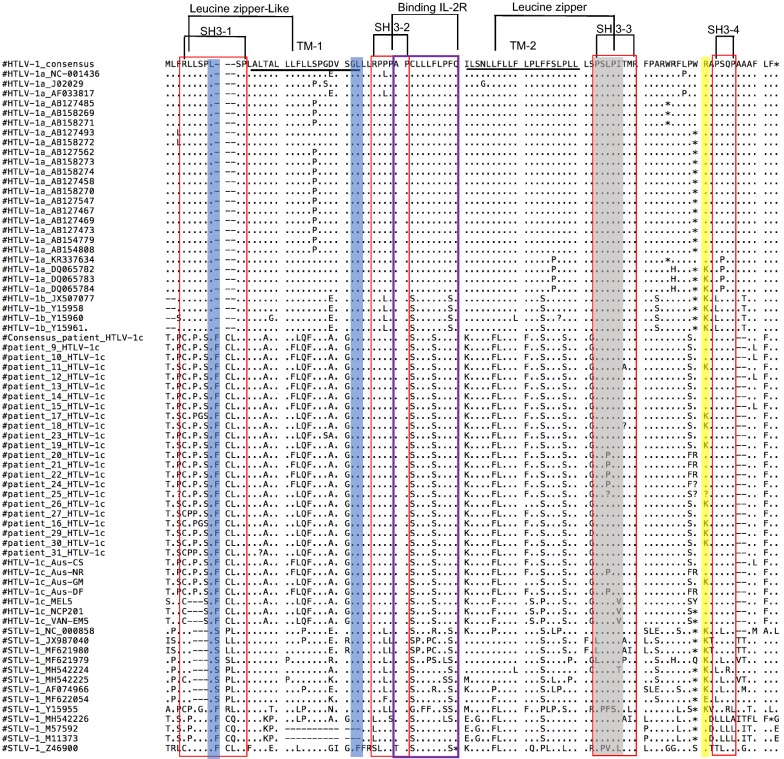
Amino acid sequence analysis of HTLV and STLV orf-I proteins. Alignment of amino acid sequences of p12 from HTLV-1A prototype (NC-001436; J02029; AF033817), and other previously described p12 sequences for HTLV-1A, HTLV-1B, HTLV-1C and STLV-1 available on Genbank. A dash (–) indicates a gap in the amino acid alignment, an asterisk (*) represents a stop codon, and a period (.) represents amino acid similarity. Functional elements are indicated as follows: the proteolytic cleavage sites between positions 9 and 10, and between 29 and 30 are highlighted in blue. The calcineurin binding motif is highlighted in gray, the ubiquitylation site is highlighted in yellow, the four SH3 binding domains are outlined in red, and IL-2Rβ and γ binding domain is outlined in magenta. The multi-alignment was performed with the Mega7 program using default parameters

Four major geographic subtypes of HTLV-1 have been identified: HTLV-1A, HTLV-1B, HTLV-1C and HTLV-1D [14, 115, 116], with HTLV-1C being the most divergent. To investigate the degree of divergence between the two HTLV-1 clades A and C, we compare three HTLV-1A representative sequences (NC-001436, J02029 and AF033817) and the seven HTLV-1C Australian and Melanesian complete genome sequences available online (GenBank KF242505, KF242506, JX891478, JX891479, KX905202, KX905203, L02534). Pairwise comparison at the nucleotide level shows higher conservation among structural genes *env*, *pol*, *pro*, *gag* compared to the regulatory genes *p30*, *p27*, and *p1*2 [117]. Interestingly, the greatest nucleotide and amino acid divergence between these two clades was observed in *orf*-*I*. In all seven HTLV-1C sequences, nucleotide substitution was present within the p12 *orf*-*I* at position 6840. This mutation leads to substitution of the start codon AUG (methionine) to ACG (threonine) in all Australian HTLV-1C isolates, and to UCG (serine) in the Melanesian isolate [118–120]. Moreover, the multiple sequence alignment of 22 HTLV-1C-infected patients [117] from an indigenous Australian cohort reveals the presence of this T6840C nucleotide substitution in 100% of the subjects (Fig. 5). Given that serine and threonine are both small polar amino acids, the different amino acid substitutions between the two clades suggests the occurrence of an evolutionary event in the isolated endemic population. In addition to this mutation, amino acid comparison demonstrated 21 significant amino acid substitutions, with 11 observed in the first 30 amino acids of the p12 that is cleaved in the endoplasmic reticulum (ER) to process the p8 isoform (Fig. 5). The significance of p12 substitutions and deletions remain unclear, but it is likely that variations within HTLV-1C p12 are implicated in its novel disease progression. Although ATLL and HAM/TSP cases have been identified in HTLV-1C-infected individuals, the majority of patients develop bronchiectasis and lung disease [24, 121]. It is important to note that since the antisense transcribed *hbz* overlaps 303 nucleotides within the *orf*-*I*, any changes in the HTLV-1C p12 coding region could also potentially affect HBZ amino acid sequence, expression, and function. Since HBZ and Tax are thought to play distinct but related roles during the multi-step oncogenesis and inflammation caused by the virus, the imbalanced expression of HBZ and Tax in HTLV-1C patients may impact its novel disease progression.

#### HTLV-1 subtype orf-I expression by alternative splicing

Alternatively, the virus may compensate for the mutation at the p12 initiating methionine to produce a protein of similar function by using an alternative splice acceptor or start site from a different gene region. The existence of different, alternative acceptor splice sites at the 3′ end of HTLV-1A have previously been described. Splice acceptor sites at position 6383, 6478, and 6875 were defined in infected cell lines and patient samples [44, 122–127]. One bicistronic mRNA, *rex*-*orf*-*I*, uses an initiation codon in exon 2 and the acceptor splice site at position 6383 to encode a protein of 152 amino acid referred to as the Rex-orf-I protein of 19 kDa. In this mRNA, the first coding exon of the Rex protein is joined in frame to p12/p8. Interestingly, ectopic expression of the of *rex*-*orf*-*I* cDNA yielded a 12-kDa protein that has the same relative migration as the p12 protein expressed by the singly spliced *orf*-*I* RNA. It has been demonstrated that this mRNA could encode p12 since the internal *orf*-*I* AUG is present in this mRNA, or p12 could be generated by posttranslational cleavage of the larger precursor protein [53, 124, 128]. Therefore, it is likely that both the doubly and singly spliced mRNAs encode the p12 protein. Moreover, the putative protein products from *rex*-*orf*-*I* show conservation of the amino acids that constitute the hallmark motifs implicated in p12 function. Thus, the protein products of *rex*-*orf*-*I* could possibly compensate for the role of p12 in viruses that do not contain the canonical initiation codon of p12 (Table 1).

**Table 1:** Variation in viral *orf*-*I* proteins

	Protein	mRNA	Size (aa)	References
HTLV-1A	p12/p8 ?	*orf*-*I*^a^ *rex*-*orf*-*I*^b^	99 152	[44, 124]
HTLV-1B	?	?	?	
HTLV-1C	p16	*rex*-*orf*-*I*^b^	153	Our unpublished data
HTLV-2	p10 p11	*rex*-*orf*-*I*^b^ *tax*-*orf*-*V*^b^	83 83	[91]
STLV-1	p12/p8	*orf*-*I*^a^	86/91	[129]
STLV-3	p9	*orf*-*I*^a^	79	[92]

? indicates that the data remain unknown

^a^Singly spliced

^b^Doubly spliced

#### orf-I genetic variation in STLV-1 subtypes

Analyses of HTLV-1 and STLV-1 viral strains from throughout the world have shown that they are closely related genetically, and they have been grouped together under the label, primate T-cell lymphotropic virus type 1 (PTLV-1). Moreover, it has been suggested that HTLV-1 has a simian origin and was originally acquired by humans through interspecies transmission from STLV-1-infected Old World monkeys. This hypothesis was supported by the high percentage of identity between STLV-1 strains from chimpanzees and mandrills with some HTLV-1 strains present in inhabitants of West and Central Africa. In these specific areas, zoonotic transmission from non-human primates (NHP) infected with STLV-1-to humans is still ongoing today [120, 130–132].

Despite its wide geographic distribution and identification in more than 20 Old World primate species in both Asia and Africa, only a few complete STLV-1 genome sequences are available [131, 133–136]. An early in vitro transcription-translation analysis of STLV-1 strains from naturally infected feral monkeys from Central and West Africa showed that STLV-1 p12 sequences exhibit interstrain genetic variability at both the nucleotide and amino acid level. Interestingly, this high variability seems to be specific to the STLV-1 p12 region since low genetic variability has been described in other genomic regions of STLV-1 [137–139]. Moreover, STLV-1 was shown to encode a 91 amino acid p12 protein in contrast to the 99 amino acid p12 protein found among HTLV-1A strains around the globe [129, 140]. The truncated STLV-1 p12 protein is the result of a change from glutamine to a stop codon, leading to a premature termination codon at amino acid residue 92, except in the STLV-1 Tan90 isolate (AF074966), where this UAG codon was found at residue 87 (Fig. 5) [129]. This feature had not been reported in HTLV-1 and was thought to be an important genetic difference between STLV-1 and HTLV-1.

### Is *orf*-*I* expression dispensable in humans?

This review has highlighted the important functions of *orf*-*I* in promoting infected cell proliferation and their evasion from host immune recognition. Further, expression of p12/p8 is necessary in both the macaque and humanized mouse models. One would therefore expect that the viral protein is important for establishing a lifelong infection in humans as well. This hypothesis was supported by a study that looked at 160 HTLV-1-infected individuals (HAM/TSP or carriers) from various geographical regions, in which none of the approximately 1600 *orf*-*I* cloned sequences analyzed had a premature stop codon [58]. However, an earlier study identified truncated p12 proteins at position 82, 87, and 91, in HAM/TSP and ATLL patients [141]. In a study analyzing p12 sequences from 144 HAM/TSP patients, 41 ATLL patients, and 46 carriers from the Kagoshima region of Japan, the authors found 8 HAM/TSP patients and 2 ATLL patients with truncated (82 aa or 87 aa) p12 proteins, for a total frequency of 4% (Fig. 5) [141].

Sequence analysis, however, shows that these truncated proteins retain the leucine zipper sites, the dileucine motif, the calcineurin binding sites, and the receptor binding sites for IL-2 beta and gamma chains and both cleavage sites (Fig. 5). As mentioned earlier, p12 contains four SH3 binding motifs. While SH3-2 and SH3-4 were demonstrated to positively regulate NFAT, SH3-1 and SH3-3 were found to exert a negative effect on NFAT activation. Thus, the premature stop codon of p12 in HTLV-1 and STLV-1 generating 82 and 87 aa sequences may negatively impact the activation of NFAT, as shown in vitro in studies by Ding et al. [142]. However, the functional consequence of these truncations is not yet known.

In the Japanese cohort analysis, the authors also found that the premature stop codon in the *orf*-*I* gene was stably maintained over years in these individuals. Moreover, they found one HAM/TSP patient in which a nucleotide substitution from G to A at position 6836 resulted in the destruction of the initiation codon of p12. This virus was also found in two of the patient’s sisters, one carrier and one with HAM/TSP, indicating maternal transmission [141] similar to the mutation previously described in STLV-1 [140]. This study did not exclude the possibility that *orf*-*I* could be expressed by an alternative doubly spliced mRNA in these patients (Table 1). It is important to note that studies that examine the p12 sequence from patient PBMCs in vivo have relied on PCR amplification and cloning of viral DNA regions, resulting in amplification of the most dominant clones [58, 59, 141] that may not be infectious. Therefore, it is possible that where premature termination of p12 was found, minor intact clones are also present that contribute to infection and/or viral persistence.

## Conclusions

The lifetime risk of developing ATLL or TSP/HAM has been estimated to be 2–5% depending on the ethnic origin of the infected individual, with a latency period between 40 and 60 years (reviewed in [143, 144]). Therefore, infected T-cells must be able to evade the host immune response to establish a persistent infection. The role of p12 and p8 in HTLV-1A pathogenesis is beginning to be uncovered, and evidence points to a central role for the *orf*-*I* protein products not only in viral transmission, but also in virus immune evasion and persistence. Sparing HTLV-1-infected cells may account for clonal expansion and contribute to disease development. Although the genomic organization of HTLV-1C closely resembles that of the cosmopolitan HTLV-1A, several differences at the nucleotide and amino acid level appear to be unique to the Australo-Melanesian HTLV-1C. Among these differences is the absence of the *orf*-*I* initiation codon in 100% of sequences from a remote Indigenous Australian cohort and in the complete HTLV-1C genome sequences now available online.

We don’t believe this observation suggests that *orf*-*I* expression is dispensable for HTLV-1C transmission and infectivity. Instead, we favor the hypothesis that an alternatively spliced mRNA may be used to provide the AUG initiation codon for the *orf*-*I* encoded protein(s). Interestingly, proteins that show p12 like functions have been identified in HTLV-2 and STLV-3 [91, 92]. Further studies are still needed to determine the role of *rex*-*orf*-*I* in infection, transmission, and pathogenesis of different HTLV-1 subtypes. Despite having had the complete genomic sequence for HTLV-1 for over 30 years, the mechanisms that govern disease status and host immune response are still unclear. We believe that developing a greater understanding of the complex kinetics, expression level, and function of the genes encoded in the 3′ end of the virus will allow us to develop novel therapeutic approaches for the treatment of HTLV-1 infection.

## Supplementary information

**Additional file 1: Figure S1.** Structure of the HTLV-1 proviral genome. The proviral DNA with the LTRs, and the unspliced, singly spliced and doubly spliced mRNA transcripts are shown. The names of the gene transcripts are depicted inside each specific box. Solid lines indicate the exons and the dotted lines indicate the introns.

## Data Availability

Not applicable.

## Abbreviations

ATLL: adult T-cell leukemia/lymphoma
β2M: β_2_ microglobulin
BLV: bovine leukemia virus
BPV: bovine papillomavirus
Ca^2+^: cytosolic calcium
CTL: cytotoxic T-lymphocyte
ER: endoplasmic reticulum
HAM/TSP: HTLV-1-associated myelopathy/tropical spastic paraparesis
Hc: heavy chain
HTLV: human T-cell leukemia virus
ICAM: intercellular adhesion molecule
IL-2: interleukin-2
IL-2R: interleukin-2 receptor
JAK-STAT: Janus-associated kinase and signal transducer and activator of transcription
LAT: linker for activation of T-cells
LFA: lymphocyte function-associated antigen
LZ: leucine zipper
MHC: major histocompatibility complex
NFAT: nuclear factor of activated T-cells
NHP: non-human primate
NK: natural killer
orf: open reading frame
PLCγ1: phospholipase C-γ-1
POM: pomalidomide
PTLV: primate T-cell lymphotropic virus
PxxP: proline-rich
SH3: Src homology 3
STAT5: signal transducer and activator of transcription 5
STLV: simian T-cell leukemia virus
TCR: T-cell receptor
TM: transmembrane
TNT: tunneling nanotube
V-ATPase: vacuolar ATPase
WT: wild type
