# Supplementary material for: Role of HTLV-1 orf-I encoded proteins in viral transmission and persistence
Source: Retrovirology. 2019 Dec 18;16:43. doi: 10.1186/s12977-019-0502-1 (PMC6921521; doi:10.1186/s12977-019-0502-1)
Supplement: Supplementary file 1 — Additional file 1: Figure S1. Structure of the HTLV-1 proviral genome. The proviral DNA with the LTRs, and the unspliced, singly spliced and doubly spliced mRNA transcripts are shown. The names of the gene transcripts are depicted inside each specific box. Solid lines indicate the exons and the dotted lines indicate the introns. [file 12977_2019_502_MOESM1_ESM.pptx]

## Slide 1
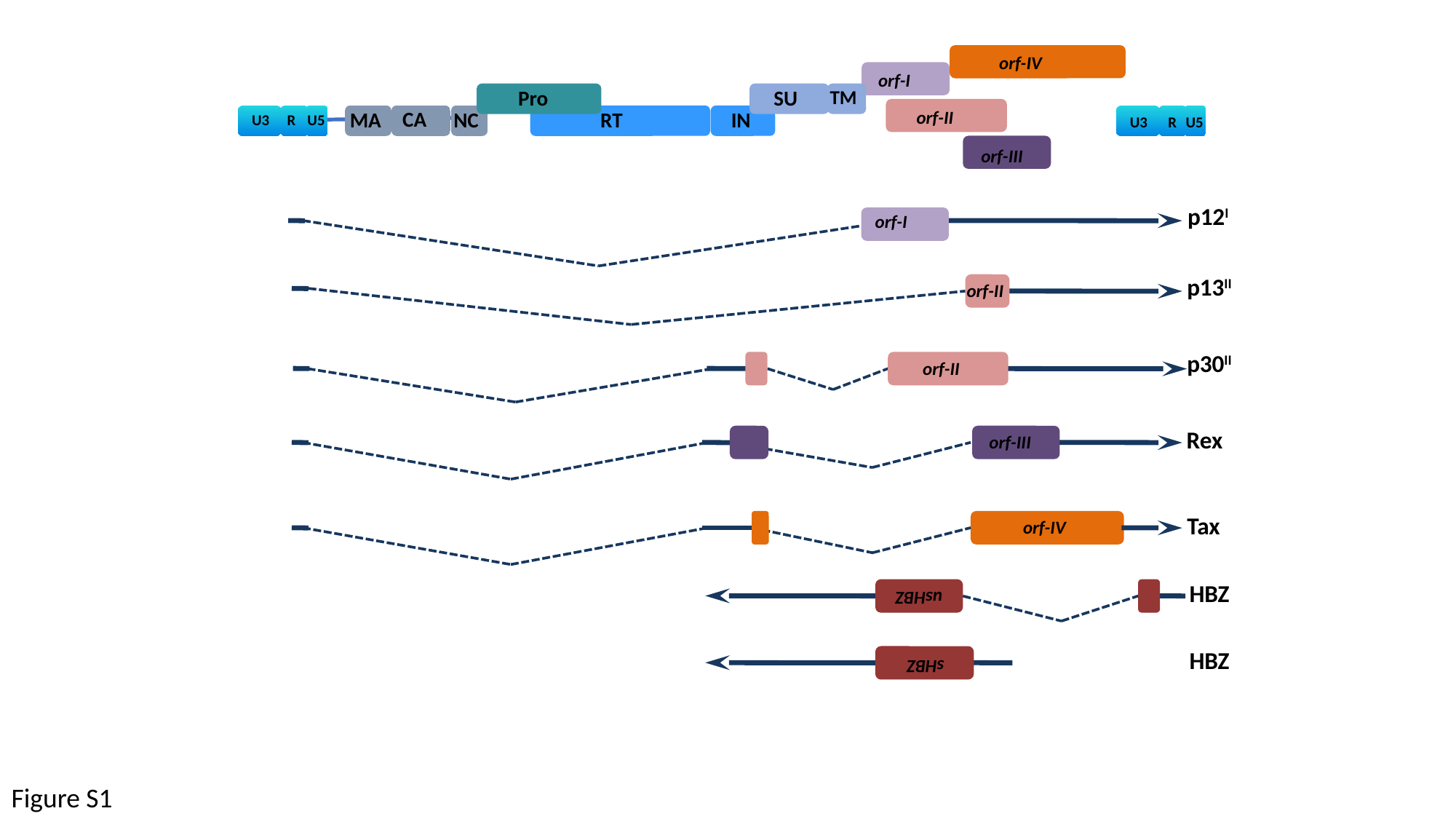

orf-IV
orf-I
Pro
SU
TM
CA
MA
NC
RT
IN
U3
R
U5
U3
R
U5
orf-II
orf-III
p12I
orf-I
p13II
orf-II
p30II
orf-II
Rex
 orf-III
Tax
orf-IV
HBZ
HBZ
usHBZ
sHBZ
Figure S1
